# Effects of Different Levels of Intraocular Stray Light on Kinetic Perimetry Findings

**DOI:** 10.1371/journal.pone.0127159

**Published:** 2015-05-12

**Authors:** Kazunori Hirasawa, Nobuyuki Shoji, Karen Isono, Manami Tsuchiya

**Affiliations:** Orthoptics and Visual Science, Department of Rehabilitation, School of Allied Health Sciences, Kitasato University, Kanagawa, Japan; Bascom Palmer Eye Institute, University of Miami School of Medicine, UNITED STATES

## Abstract

**Purpose:**

To evaluate the effect of different levels of intraocular stray light on kinetic perimetry findings.

**Methods:**

Twenty-five eyes of 25 healthy young participants were examined by automated kinetic perimetry (Octopus 900) using Goldmann stimuli III4e, I4e, I3e, I2e, and I1e. Each stimulus was presented with a velocity of 3°/s at 24 meridians with 15° intervals. Four levels of intraocular stray light were induced using non-white opacity filter (WOF) filters and WOFs applied to the clear plastic eye covers of the participants. The visual acuity, pupil diameter, isopter area, and kinetic sensitivity of each meridian were analyzed for each WOF density.

**Results:**

Visual acuity deteriorated with increasing WOF densities (p < 0.01). With a visual acuity of 0.1 LogMAR units, the isopter areas for III4e, I4e, I3e, I2e, and I1e decreased by -32.7 degree^2^ (-0.2%), -255.7 degree^2^ (-2.6%), -381.2 degree^2^ (-6.2%), -314.8 degree^2^ (-12.8%), and -59.2 degree^2^ (-15.2%), respectively; kinetic sensitivity for those stimuli decreased by -0.1 degree (-0.1%), -0.8 degree (-1.4%), -1.6 degree (-3.7%), -2.7 degree (-9.7%), and -1.7 degree (-16.2%), respectively. The pupil diameter with each WOF density was not significantly different.

**Conclusion:**

Kinetic perimetry measurements with a high-intensity stimulus (i.e., III4e) were unaffected by intraocular stray light. In contrast, measurements with the I4e, I3e, I2e, and I1e stimuli, especially I2e and I1e, were affected. Changes in the shape of the isopter resulting from opacity must be monitored, especially in cases of smaller and lower-intensity stimuli.

## Introduction

Kinetic perimetry is generally performed using the Goldmann perimeter[1–3], which requires the examiner to manually control the moving stimulus. Therefore, inherent examiner bias, based on variable skills of the examiners, reduces the accuracy and consistency of manual kinetic perimetry findings [4]. To remove this bias, a few automated kinetic perimeters have been developed[5–8]. Using automated kinetic perimeters, researchers have been able to examine the optimal stimulus velocity[9], learning effect, and repeatability in normal participants[10], and to determine the effect of optical defocus on kinetic perimetry findings[11].

Kinetic perimetry has been generally performed in extensive regions from central to peripheral areas using various stimulus sizes and intensities for diagnosis and follow-up of glaucoma patients and individuals with other ocular and neurologic disorders affecting the visual pathways. When cataracts coexist with these diseases, changes in the visual field defects may indicate deterioration of the condition or cataract, leading to confusion. In such cases, it is important to evaluate the effect of opacity on kinetic perimetry findings. Many studies have reported a decrease in sensitivity caused by ocular media opacity or induced intraocular stray light measured by standard automated perimetry[12–18], frequency doubling technology[16,17,19–21], or short wave-length automated perimetry[13,17,22,23] within 30° of the visual field. However, few studies have examined the effects of opacity on kinetic perimetry findings[24,25]. Moreover, manual kinetic perimetry was used to obtain perimetry findings in the aforementioned studies[24,25]. Therefore, further investigation using automated kinetic perimetry is warranted.

The aim of this study was to evaluate the effects of intraocular stray light induced by a white opacity filter (WOF) on kinetic perimetry findings in healthy participants using automated kinetic perimetry.

## Methods

Twenty-five healthy young participants (3 men) with a mean age of 22.0 ± 3.2 years and mean spherical equivalent of -3.58 ± 2.29 diopter who had undergone automated kinetic perimetry examinations and gave informed consent were recruited in this prospective observational case series study. This study followed the tenets of the Declaration of Helsinki. Each participant provided written informed consent after the ethics committee of Kitasato University School of Allied Health Science approved the study (No. 2013–26).

All participants underwent comprehensive ophthalmic examinations, including noncycloplegic refraction testing, visual acuity (VA) testing at 5 meters using a Landolt ring chart, intraocular pressure (IOP) measurement, ocular axial length measurement, and slit-lamp and fundus examination by a glaucoma specialist (NS). Participants who had a corrected VA of 20/20 or better, IOP of 21 mmHg or less, normal optic disc appearance, and no ophthalmic diseases that affected the visual field test were included in this study. The eye with a lower amount of astigmatism was selected as the study eye. If the level of astigmatism was the same in both eyes, the eye with lower myopia was chosen as the study eye.

**Fig 1:**
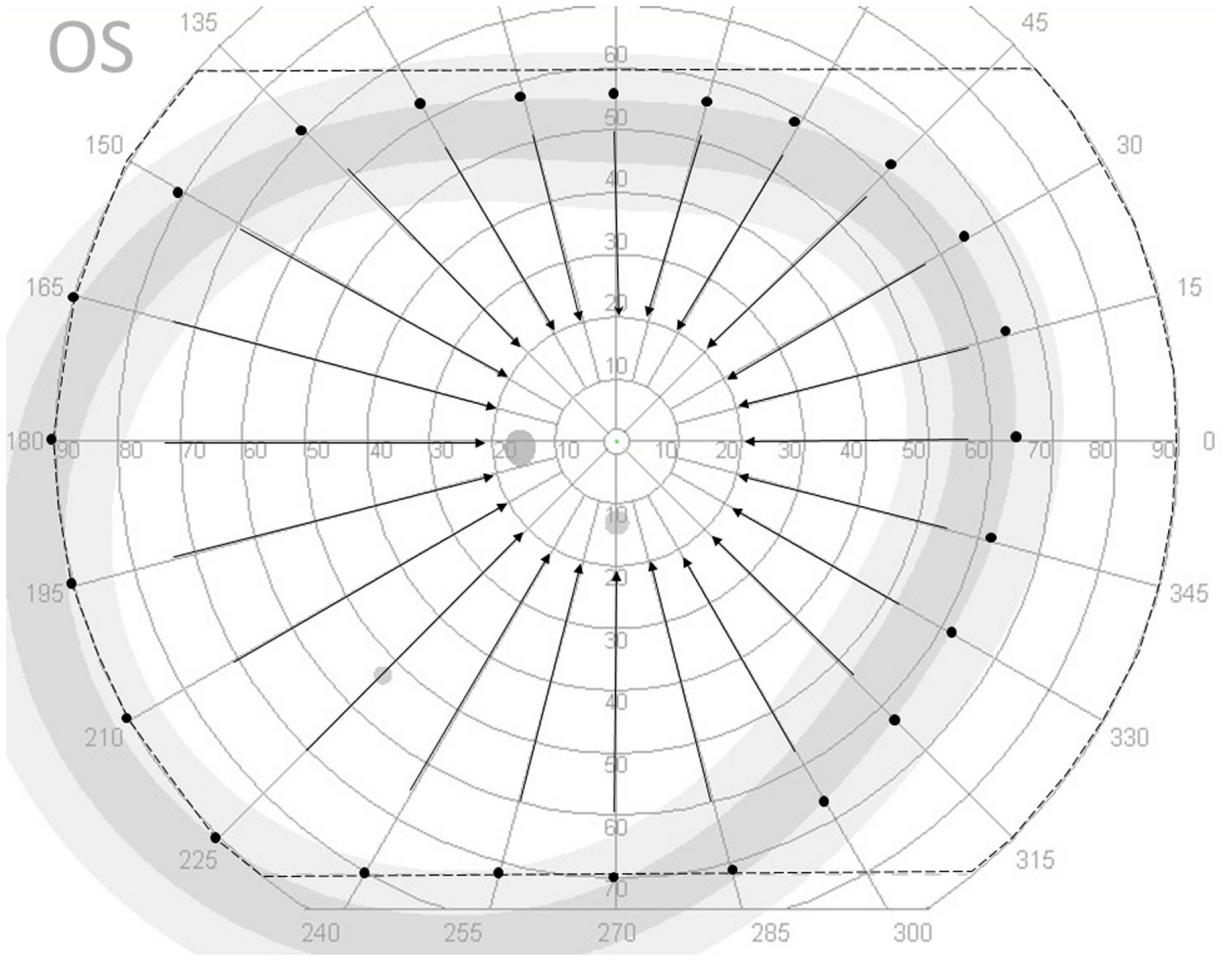
Measurable area and starting locations with a moving stimulus at each meridian. The Octopus 900 perimeter device can measure the area outlined by the dashed line. The starting locations with a moving stimulus at each meridian are depicted using the III4e stimulus as an example. The stimulus is presented randomly on each meridian from the extreme periphery of normal age-corrected kinetic sensitivity to the center. If the normal age-corrected kinetic sensitivity is outside the measurable area (dashed line), the starting location is set to the extreme end of the measurable area on the same meridian. The I4e, I3e, I2e, and I1e stimuli were also measured using the same method.

Kinetic perimetry was performed using an Octopus 900 automated kinetic perimeter measurement device (Haag-Streit, Koeniz, Switzerland). The measurement conditions for automated kinetic perimetry were calibrated automatically to the same measurements used for the Goldmann perimeter with a background luminance of 10 cd/m^2^ (31.4 asb). Goldmann stimulus sizes and intensities of III4e, I4e, I3e, I2e, and I1e were used for measurement. The stimulus velocity was 3°/sec based on a previous investigation[9], and the stimuli were presented in the following order: III4e, I4e, I3e, I2e, and I1e. However, the starting locations with a moving stimulus were presented randomly for each stimulus. Fig 1 shows the measurable area of the perimeter and the starting locations with a moving stimulus. The stimulus test locations were 120 predetermined points, with each stimulus applied to 24 points with a 15 degree meridional interval. The stimuli were presented randomly from the extreme periphery of the normal age-corrected kinetic sensitivity to the center. The fixation of each participant in this study was monitored using a display as described in previous reports[9,11,26–30]. Although the Octopus 900 Perimeter device adjusts for reaction time by adjusting the isopter area according to the response time for the stimulus presentation, we did not adjust the reaction time in this study because kinetic sensitivity with each density of opacity was not compared between participants (inter-participant); it was only evaluated for each participant (intra-participant).

**Fig 2:**
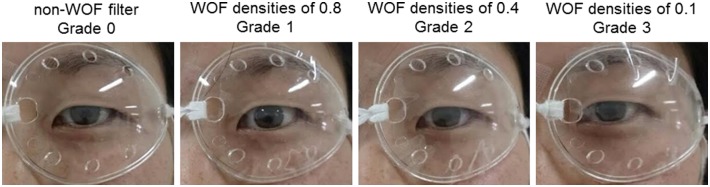
Sealed clear plastic eye cover with applied white opacity filters (WOFs). From left to right, non-WOF filter (Grade 0) and WOF densities of 0.8 (Grade 1), 0.4 (Grade 2), and 0.1 (Grade 3) are presented.

Intraocular stray light was induced by WOFs with a density of 0.8, 0.4, and 0.1 (Bangerter occlusion filter; Ryser Optik AG, St Gallen, Switzerland)[31]. A lower filter density indicates higher opacity. Participants underwent the automated kinetic perimetry examination by wearing sealed plastic eyes (Fig 2). Each WOF was applied to the sealed clear plastic eye cover (eye cup; Nagoya Spectacle Co., Inc., Aichi, Japan), and a clear plastic eye cover sealed without a WOF was also examined for comparison. Then, non-WOF and WOF densities of 0.8, 0.4, and 0.1 were defined as Grade 0, Grade 1, Grade 2, and Grade 3, respectively.

One eye of each participant was tested. All participants underwent automated kinetic perimetry with Grade 0 as the baseline at the beginning, and WOF densities of Grade 1, Grade 2, and Grade 3 were measured in a random order four times a day. Participants were allowed to rest for at least 5 to 10 min between sessions. The refraction of the participants was corrected to near vision (30 cm) using disposable soft contact lenses.

The main outcome measures were change in visual acuity, pupil diameter, isopter area, and kinetic sensitivity at each meridian while wearing the plastic eye sealed with each WOF. In addition, changes in isopter area and kinetic sensitivity at each meridian per 0.1 LogMAR unit were also calculated at each stimulus. The VA test was also performed with the participants wearing the plastic eye sealed with each WOF before each measurement. The pupil size was measured by capturing a screenshot of the pupil image on the display screen of the Octopus 900 perimeter before each measurement. The isopter area was evaluated according to the value that was automatically calculated by the Octopus 900 perimeter after measurement, and the kinetic sensitivity at each meridian was evaluated at the position from which the participant recognized the stimulus, presented peripherally, without moving their eyes. Left-eye meridians were evaluated as mirror images to enable concurrent analysis of data from the left and right eyes. Data were excluded from analyses if fixation loss occurred, or if the corrective contact lenses fit poorly.

### Statistical analysis

All data were compiled in Microsoft Excel and analyzed using the statistical software packages SPSS version 21.0 (IBM Japan, Ltd., Tokyo, Japan) and G*Power3 version 3.1.7 (Franz Faul, Universität Kiel, Germany). The current Octopus 900 perimeter system does not display the coordinate axes for expressing the kinetic sensitivity, and it does not measure the pupil size. Therefore, the kinetic sensitivity was calculated in degrees from the fixation point, and pupil size was measured using ImageJ version 1.47 (Wayne Rasband, National Institutes of Health, Bethesda, MD). The decrease in isopter area and kinetic sensitivity at each meridian per 0.1 LogMAR unit was calculated using linear regression analysis for each participant, and the slope for each participant was averaged for each stimulus.

The normality of the data distribution was evaluated using the Shapiro-Wilk test. Repeated measures analysis of variance and post hoc testing with Dunnett’s test or Wilcoxon signed-rank test with the Bonferroni correction for the number of tested variables were used to compare the visual acuity, pupil diameter, isopter area, and kinetic sensitivity between the participants without WOF (Grade 0) and those with WOF densities (Grades 1, 2, and 3). VA was calculated in LogMAR. The effect size, α error, power (1-β error), and nonsphericity correction were 0.25, 0.05, 0.80, and 0.25, respectively, and the required sample size was 21 participants for the four repeated measurements[32].

## Results

No participants were excluded by the exclusion criteria. Thus, 25 eyes of 25 participants were analyzed. Table 1 shows the demographic data for the participants. The participants included 3 men and 22 women, with a mean age of 22.0 ± 3.2 years, mean spherical equivalent of -3.58 ± 2.29 diopter, mean VA (LogMAR) of -0.14 ± 0.08, mean IOP 13.1 ± 2.0 mmHg, and mean axial length of 24.74 ± 1.05 mm

**Table 1:** Demographic data and ocular characteristics of the participants.

Parameter	Mean ± Standard deviation	Range (min to max)
Spherical power (diopter)	-3.27 ± 2.35	-10.00 to 0.00
Astigmatic power (diopter)	-0.61 ± 0.38	-1.50 to 0.00
Spherical equivalent (diopter)	-3.58 ± 2.29	-10.13 to -0.38
Visual acuity (logMAR)	-0.14 ± 0.08	-0.28 to 0.04
Intraocular pressure (mmHg)	12.9± 2.1	9.3 ± 18.0
Axial length (mm)	24.74 ± 1.02	22.72 to 26.84

**Fig 3:**
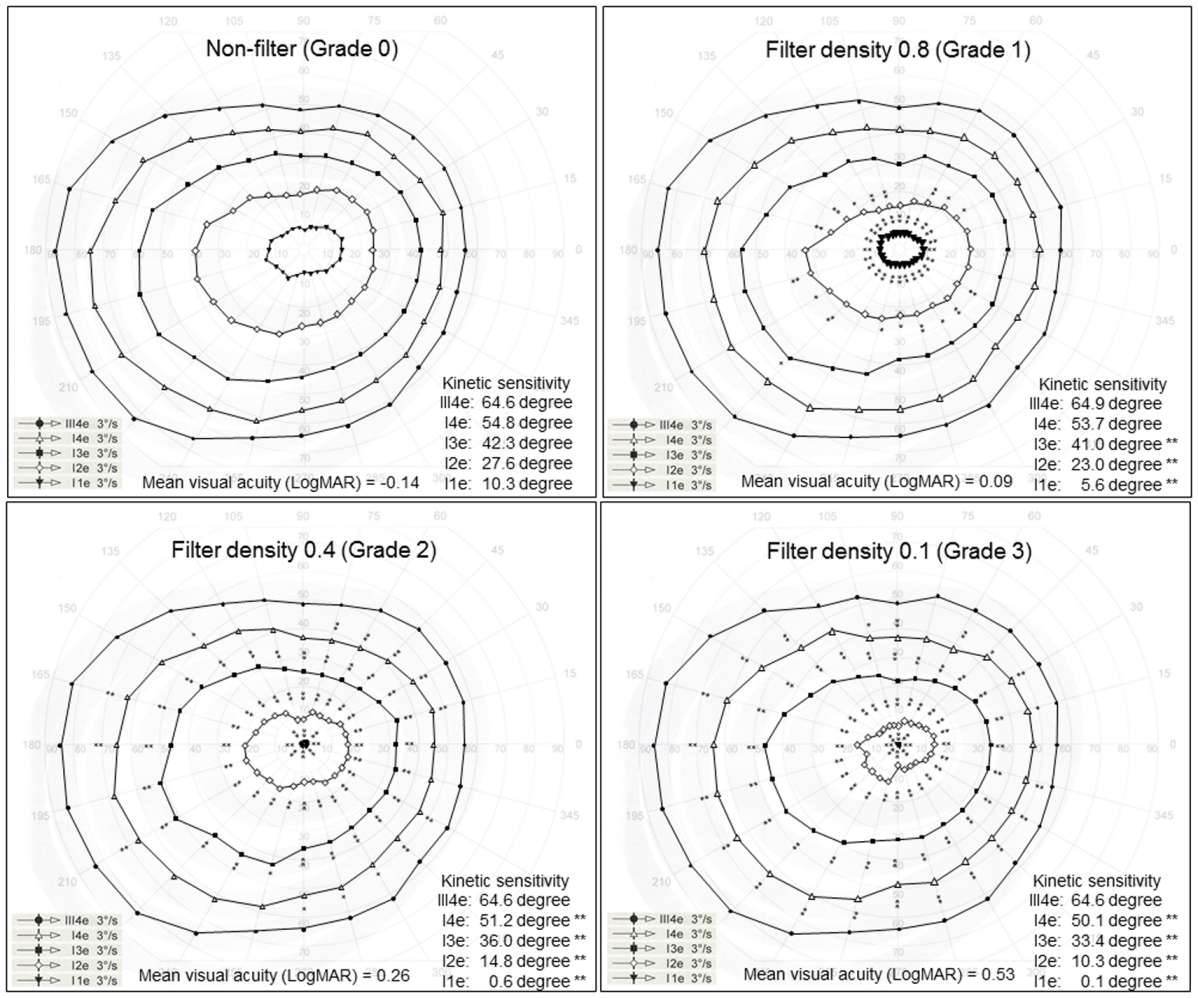
Kinetic sensitivity at each meridian of each stimulus under Grades 0 to 3. Each plot was expressed as an average value for all participants. The mean visual acuity (logarithmic minimum angle of resolution, LogMAR) and mean kinetic sensitivity measured for each stimulus are shown in the lower middle and right, respectively. The symbols * and ** indicate that the kinetic sensitivity significantly decreased with p < 0.05 and 0.01 compared to the baseline Grade 0, respectively.

Table 2 shows the changes in the visual acuity, pupil diameter, and isopter area with each WOF density. VA with each WOF significantly decreased as WOF density increased compared to Grade 0 (respective p < 0.05). In contrast, the pupil diameter with each WOF density was not significantly different from that at Grade 0. Although the isopter area of III4e with each WOF density was not significantly different from that with Grade 0, the isopter area of I4e, I3e, I2e, and I1e with each WOF density significantly decreased with increasing filter density compared to that with Grade 0 (respective p < 0.05). Fig 3 shows the kinetic sensitivity at each meridian of each stimulus between Grades 0 and 3. The kinetic sensitivity at each meridian of each stimulus under each filter density was similar to the isopter area.

**Table 2:** Changes in the visual acuity, pupil diameter, and isopter area under each white opacity filter density.

	White opacity filter density			
	Grade 0	Grade 1	Grade 2	Grade 3
Visual acuity (LogMAR)	-0.14 ± 0.08	0.09 ± 0.10**	0.26 ± 0.12**	0.53 ± 0.14**
Pupil diameter (mm)	5.8 ± 1.1	5.6 ± 0.8	5.8 ± 0.9	5.5 ± 0.9
Isopter area (degree^2^)				
III4e	13366.4 ± 1200.8	13431.1 ± 1040.2 (0.7 ± 4.0%)	13236.4 ± 1092.9(-0.8 ± 4.1%)	13243.2 ± 1092.9(-0.7 ± 4.7%)
I4e	9589.4 ± 844.4	9183.6 ± 851.6 (-4.0 ± 6.1%)	8374.8 ± 879.4*(-12.6 ± 5.7%)	7967.5 ± 819.6**(-16.7 ± 6.8%)
I3e	6144.5 ± 772.1	5419.6 ± 906.1**(-11.5 ± 12.2%)	4244.8 ± 863.5**(-30.7 ± 12.3%)	3683.5 ± 956.6**(-40.0 ± 13.7%)
I2e	2470.7 ± 500.5	1762.0 ± 600.3**(-28.9 ± 20.8%)	819.3 ± 482.8**(-67.0 ± 18.8%)	438.0 ± 322.5**(-82.4 ± 13.3%)
I1e	376.4 ± 228.6	129.3 ± 117.9**(-60.2 ± 29.3%)	5.1 ± 12.8**(-98.5 ± 4.5%)	0.2 ± 1.1**(-100.0 ± 0.1%)

Data are given as the mean ± standard deviation and mean percent change.

LogMAR: logarithmic minimum angle of resolution.

*and ** indicate that the isopter area significantly decreased with p < 0.05 and 0.01 compared to Grade 0, respectively.

**Fig 4:**
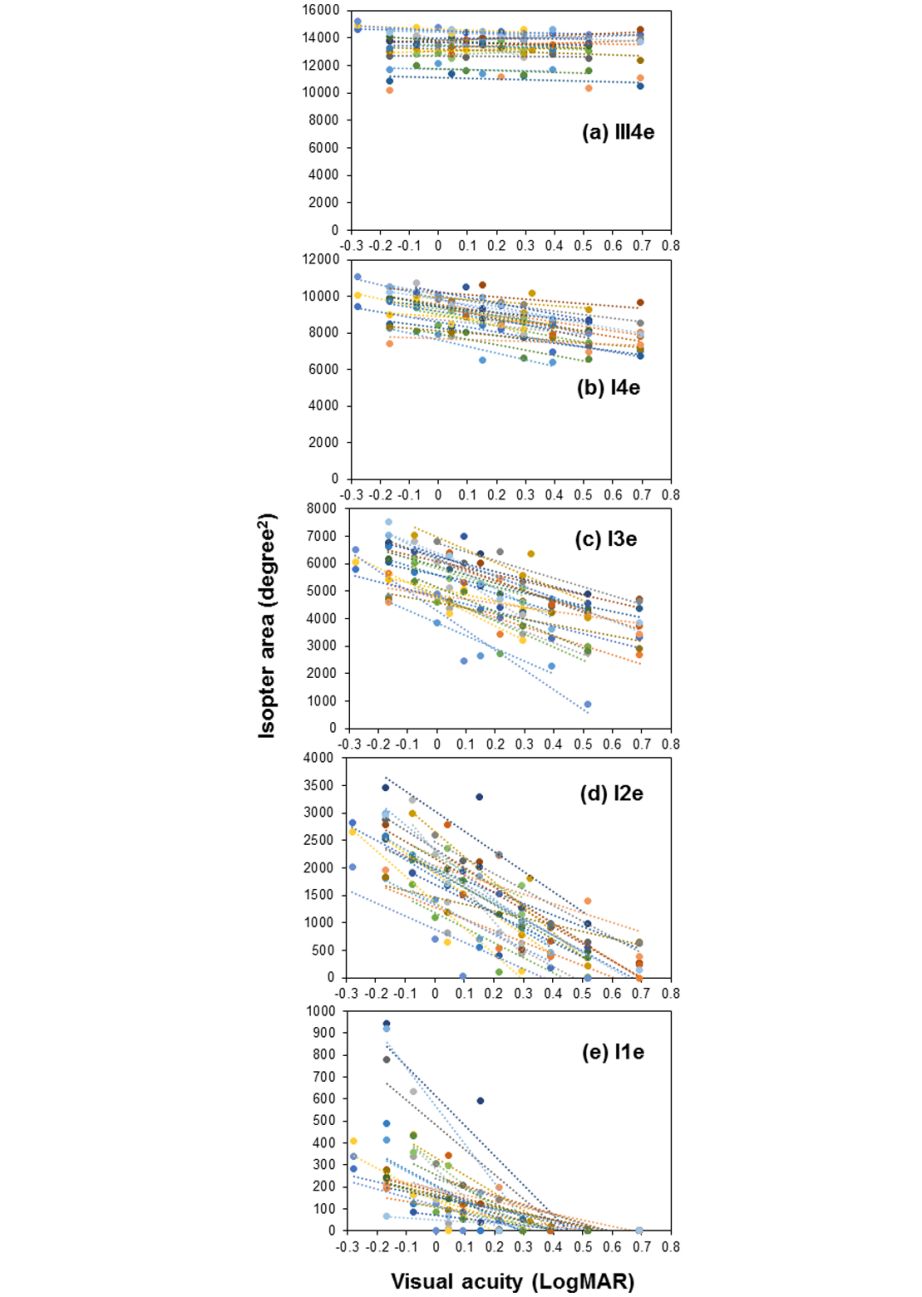
Deterioration in the isopter area per 0.1 LogMAR unit at each stimulus. Colored dots indicate deterioration in the isopter area per 0.1 LogMAR unit for each participant and the dotted lines indicate approximate straight lines.

With decreasing VA for every 0.1 LogMAR units, the isopter area of III4e, I4e, I3e, I2e, and I1e decreased by -32.7 degree^2^ (-0.2%), -255.7 degree^2^ (-2.6%), -381.2 degree^2^ (-6.2%), -314.8 degree^2^ (-12.7%), and -59.2 degree^2^ (-15.2%), respectively (Fig 4), and the kinetic sensitivity for those stimuli decreased by -0.1 degree (-0.1%), -0.8 degree (-1.4%), -1.6 degree (-3.7%), -2.7 degree (-9.7%), and -1.7 degree (-16.2%), respectively.

## Discussion

In the current study, VA (LogMAR) with each WOF decreased as the filter density increased. A previous study[33], which investigated the effect of WOF on distance VA in healthy young participants aged 22–35 years, showed that WOF densities of 0.8, 0.4, and 0.1 decreased the VA to 0.23, 0.28, and 0.93, respectively. These values were slightly different from those obtained in the current study. In the previous study[33], WOFs were applied to trial plano lenses, whereas, in the current study, WOFs were applied to sealed round clear plastic eyes. The difference in VA with each WOF density could be attributed to the magnitude of forward scatter resulting from the difference in vertex distance between the anterior surface of the cornea and WOF and the difference in form between plano lenses and sealed plastic eyes.

In the current study, the pupil diameter was not significantly different among the WOF densities. It is known that pupil dilation leads to an increase in spherical aberration as the parallel rays of incident light do not converge at the same point after passing through the lens; conversely, pupil miosis leads to an increase in diffraction as the light rays are no longer straight when they pass through the round edge of the pupil’s margin[34]. It is thought that aberration and diffraction associated with changes in pupil diameter have a limited impact on isopter area and kinetic sensitivity, as described later.

In the current study, the isopter area of III4e was not significantly affected by WOF density and that of I4e slightly decreased at dense WOFs (Grade 2 and 3), whereas those of I3e, I2e, and I1e decreased with slight increases in WOF density (Grades 1 to 3). A previous study using manual Goldmann kinetic perimetry showed that the isopter area decreased as the intensity and size of the stimuli decreased, especially for I2e [24]. Although the type of defocus was different because of WOF and optical defocus, a previous study reported that I3e, I2e, and I1e, especially I2e and I1e, were affected by optical defocus[11]. Our study obtained a similar finding. As the WOF density or cataract grade increases, intraocular stray light increases[16–18,35,36], and point spread function deteriorates. As a result, the degradation of sharp edges comprises a larger percentage of the total area of small and dim targets and comprises a smaller percentage for larger, more intense targets. Consequently, the retinal sensitivity for stimuli of lower intensity, particularly I2e and I1e, may be decreased. Because of the effect of stimulus size and intensity, the isopter area of III4e was not significantly affected by WOF density, whereas that of I4e slightly decreased at dense WOFs.

The deterioration in the isopter area and kinetic sensitivity per 0.1 LogMAR unit at each meridian was also calculated for each stimulus in this study. A previous study reported that deterioration of sensitivity, expressed as decibels, could be measured with standard automated perimetry, frequency-doubling technology, and short wave-length automated perimetry within 30° of the visual field per log stray light value[16–18]. At our institute, however, we could not investigate the decrease in sensitivity per log stray light because of the lack of a device to measure stray light. Therefore, the deterioration in isopter area and kinetic sensitivity per 0.1 LogMAR unit was calculated. Although it would have been useful to compare the findings of this study with those of previous studies[16–18], we also consider that it is important for examiners to determine the deterioration in sensitivity associated with a decrease in VA. Observation of decreased isopter area and kinetic sensitivity at each meridian per 0.1 LogMAR unit could be helpful in clinical settings.

This study has the following limitations. First, WOFs were used to induce opacity, and only young participants were included in this study. In clinical settings, the crystalline lens of a patient with cataracts is colored yellow. Therefore, the retinal sensitivity for white stimuli between patients with cataracts and young participants as in the current study would be different. Second, the overall opacity was induced by the WOF. A previous study reported that opacities situated in the posterior layers of the crystalline lens cause a defect in the visual field on the opposite side. Therefore, the results of the current study cannot be readily adapted to all cataract patients. Third, although reaction time adjustment was not performed in the current study, isopter area may change through adjustment for reaction time across different levels of WOFs because a previous study reported that reaction time changed with different diseases, particularly in glaucoma[27] and especially in the advanced disease stages[28,36]. Further investigation regarding these effects is necessary.

## Conclusion

The high-intensity III4e stimulus was unaffected by intraocular stray light. In contrast, measurements with the I4e, I3e, I2e, and I1e stimuli were affected, particularly those with the I2e and I1e stimuli. Care must be taken when the shape of isopter changes because of the opacity, especially in cases of smaller and lower-intensity stimuli.

## Supporting Information

S1 File Analyzed data. (XLSX)
